# One size does not fit all: Insights for engaging front‐line clinicians in pragmatic clinical trials

**DOI:** 10.1002/lrh2.10248

**Published:** 2020-10-13

**Authors:** Ellen Tambor, Rachael Moloney, Sarah M. Greene

**Affiliations:** ^1^ Center for Medical Technology Policy Baltimore Maryland USA; ^2^ SG Strategies Seattle Washington State USA

**Keywords:** clinician engagement, learning health systems, pragmatic clinical trials

## Abstract

**Introduction:**

Despite the proliferation of pragmatic clinical trials (PCTs) conducted in health care delivery settings, we know relatively little about how practicing clinicians perceive their potential roles in such research. Empirical evidence and practical guidance concerning clinician engagement in research is needed to inform the design and successful implementation of PCTs.

**Methods:**

We conducted a two‐phase qualitative study to better understand how and to what extent practicing clinicians should be involved in PCTs and to develop guidance for researchers on engaging front‐line clinicians in PCTs. In phase one, clinicians who spend the majority of their time providing direct patient care participated in 90‐min focus groups. In phase two, we conducted key informant interviews with PCT research teams and clinicians participating in the ADAPTABLE (Aspirin Dosing: A Patient‐centric Trial Assessing Benefits and Long‐Term Effectiveness) trial.

**Results:**

Thirty‐four physicians, nurses, and other care providers from four health care delivery organizations participated in focus groups. Focus group participants stressed the importance of engaging clinicians early in the PCT planning process to identify clinically relevant study questions, provide input on study design, and customize study protocols to fit unique clinic workflows. We conducted 18 interviews with principal investigators, project managers, and clinicians involved in the ADAPTABLE trial across six clinical data research networks. Study team members described trying multiple approaches to optimize in‐clinic recruitment and enrollment of eligible patients. Successful strategies involved several key factors related to research team interactions with eligible patients, clinicians, and clinic staff.

**Conclusions:**

More active involvement by a range of clinical stakeholders in PCT planning may help researchers avoid common barriers to trial implementation. We propose a “medium‐touch” approach to involving clinicians in PCT recruitment and enrollment that focuses clinician effort where it is most critical—to reassure eligible patients that trial participation is a safe alternative for them.

## 1 INTRODUCTION

The past decade has seen a proliferation of pragmatic clinical trials (PCTs) conducted in health care delivery settings. PCTs can be more informative than traditional randomized clinical trials because they attempt to answer questions related to the effectiveness of therapies in the “real world.”^1^ However, given the proximity of PCTs to the point of care, they require some level of involvement by healthcare professionals. A key lesson from the first cohort of PCT demonstration projects funded through the National Institutes of Health (NIH) Health Care Systems Research Collaboratory is that engagement and buy‐in at the organizational level is necessary but not sufficient for successful PCT implementation.^2^

Despite the importance of practicing clinicians to research conducted in the context of health care delivery, we know surprisingly little about how they perceive their roles in such research. A 2017 National Academy of Medicine (NAM) paper notes that the current health care ecosystem contains numerous impediments to clinician participation in learning activities, including misalignment of priorities, productivity pressures, and lack of training and incentives.^3^ Studies assessing clinician perspectives on research participation have reported generally favorable views toward research but also identify multiple barriers to participation, with lack of time and competing priorities topping the list.^4^, ^5^ Common motivations for clinicians to participate in research include acquiring knowledge to improve patient care, contributing to scientific knowledge, and professional development.^4^, ^6^, ^7^

Previous efforts to engage front‐line clinicians in research activities have suggested a range of strategies for improving engagement. For example, the importance of selecting topics of mutual interest to researchers and clinicians is frequently highlighted.^8^, ^9^, ^10^ Other recommendations for improving clinician engagement include involving clinicians in adapting study protocols to fit the practice environment, procedures, and workflow^11^, ^12^; providing comprehensive and ongoing training;^13^ identifying site‐level study champions;^12^ and recognizing individual study contributions.^14^, ^15^

Empirical evidence and practical guidance concerning clinician engagement in research remain limited and there is a fundamental gap in understanding the extent to which practicing clinicians should be involved in PCTs. We conducted a qualitative study to better understand clinician views on their potential role in research conducted in the context of health care delivery and to develop guidance for researchers on engaging front‐line clinicians in PCTs. In phase one, we conducted focus groups with clinicians who spend the majority of their time providing direct patient care and have limited research experience. Phase two involved key informant interviews with PCT research teams and clinicians participating in the ADAPTABLE (Aspirin Dosing: A Patient‐centric Trial Assessing Benefits and Long‐Term Effectiveness) trial.^16^, ^17^

## 2 METHODS

### 2.1 Focus groups

#### 2.1.1 Overview

A focus group approach was chosen for this phase of the project so that participants would have the opportunity to hear perspectives from their peers that they may not have otherwise considered. We identified focus group sites that were diverse with regard to the type of health system.

### 2.2 Participants

Four organizations agreed to participate in focus groups, including: (a) *Clinical Director's Network, Inc*. (CDN)—a practice‐based research network (PBRN) in New York City; (b) *AllianceChicago*—a network of community health centers serving primarily low‐income and uninsured patients; (c) *Edward Hines Jr. VA Hospital*—a Veterans Health Administration hospital serving veterans in the Chicago area; and (d) *Rush University Medical Center*—an academic medical center located in Chicago, IL.

The project advisory committee included representatives from each of the participating sites who, along with designated site coordinators, assisted with recruitment and meeting logistics. Invitations to participate in a 90‐min focus group were distributed via multiple channels (newsletters, email blasts, fliers). Physicians, nurses, nurse practitioners, physician assistants, and related professionals who spend the majority of their professional time providing direct patient care were invited to participate. Participants were offered $100 honoraria (except for one site where this was not permitted) and were provided a meal appropriate for the time of each group.

#### 2.2.1 Procedures

Participants completed informed consent upon arrival followed by a brief, anonymous questionnaire intended to characterize the group in terms of professional roles and research experience. Focus groups were audio‐recorded and transcribed for qualitative analysis.

Discussions were led by experienced facilitators using a discussion guide with questions and probes that were flexible enough to adapt to the flow of conversation within each group. Following an introduction to the goals of the study, participants were asked a series of questions pertaining to the roles that clinicians are sometimes asked to play in comparative effectiveness and patient‐centered outcomes research (CER/PCOR) studies.

Based on the assumption that participants may have had very limited research exposure, we began by discussing quality improvement initiatives before transitioning to questions about hypothesis‐driven research and the difference between traditional randomized clinical trials and pragmatic trials. The remainder of the discussion focused on clinician involvement across the phases of pragmatic trials.

**FIGURE 1:**
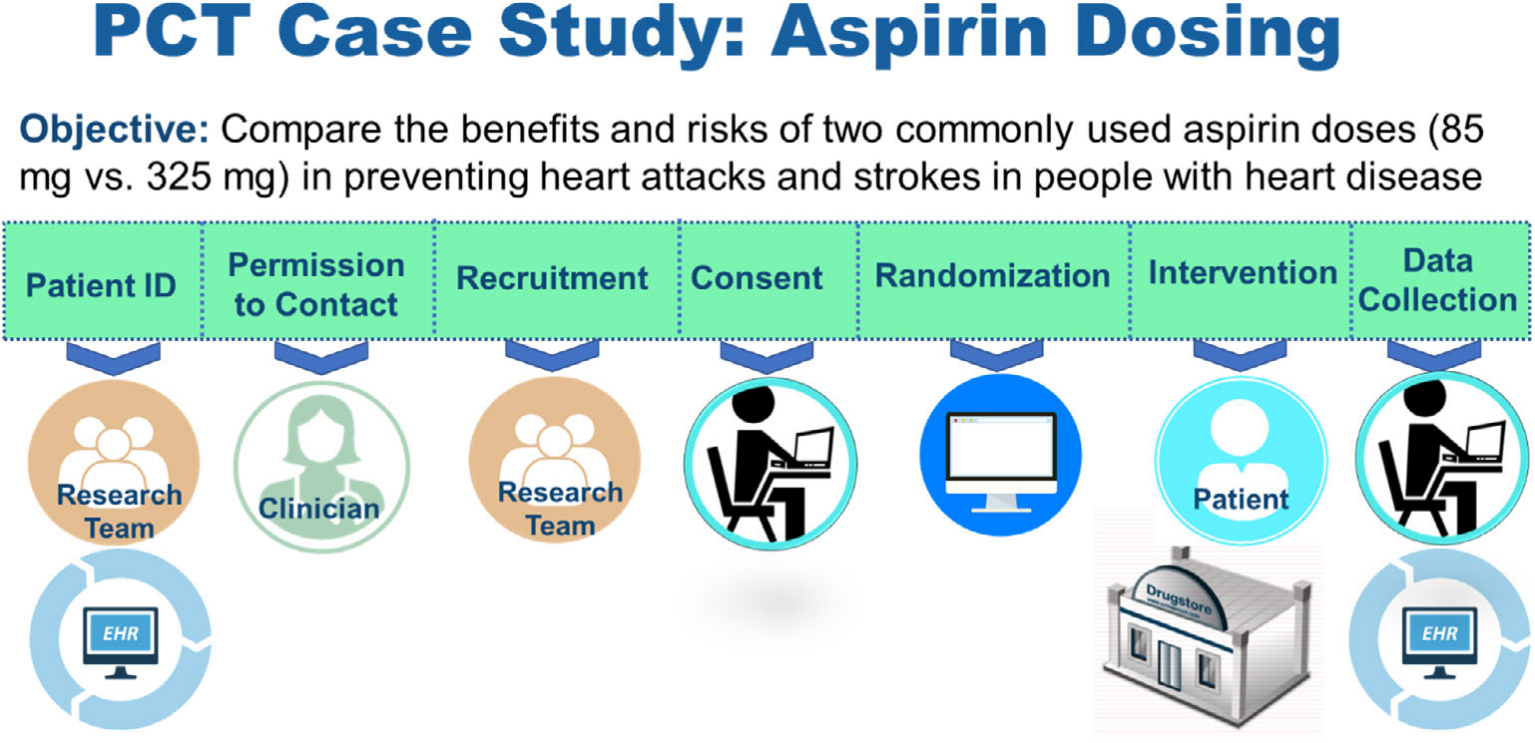
Pragmatic clinical trial case study

To facilitate a more in‐depth discussion around pragmatic trial implementation, we introduced a hypothetical case study modeled after the ADAPTABLE trial. As shown in Figure 1, the roles of patients, clinicians, and researchers in the study were described as: (a) research team identifies eligible patients using EHR, (b) clinician provides permission to contact eligible patients, (c) research team recruits and enrolls eligible patients, (d) patient completes informed consent process and is randomized via patient portal, (e) patient obtains prescribed aspirin dose and takes as instructed, and (f) data is collected from EHR by research team and from patients via the portal. After reacting to this initial protocol, participants were asked to consider how their opinions might change with various modifications.

### 2.3 Interviews

#### 2.3.1 Overview

The goal of this project phase was to examine clinician engagement in the context of a real‐life pragmatic trial. The timing of our project offered a valuable opportunity to learn from the ADAPTABLE trial. ADAPTABLE, the first pragmatic trial to leverage PCORI's National Patient‐Centered Clinical Research Network (PCORnet), aims to identify the optimal dose of aspirin for secondary prevention in patients with atherosclerotic cardiovascular disease.

### 2.4 Participants

The Mid‐South and REACHnet CDRNs agreed to participate in our project during the proposal process and additional CDRNs were invited once the project was underway.

For each participating CDRN, we invited members of the ADAPTABLE study team, including CDRN‐ and site‐level investigators and project managers, to participate in interviews. In addition, we asked study team members to help identify clinicians who might be willing to participate in brief interviews.

#### 2.4.1 Procedures

One‐hour, semi‐structured telephone interviews with ADAPTABLE team members were conducted by experienced members of the project team and, except for one group of investigators, were one‐on‐one. Interviews were audio‐recorded and transcribed for qualitative analysis.

The discussion guide included open‐ended questions regarding experience with the ADAPTABLE trial and directed questions regarding the role of clinicians in the trial and specific challenges related to clinician engagement.

Twenty‐minute semi‐structured telephone interviews with participating clinicians included a series of open‐ended questions regarding their experience with the trial.

### 2.5 Qualitative analysis

De‐identified transcripts were checked for accuracy and uploaded to NVivo 12.

Two study team members independently coded a subset of focus group transcripts using a codebook based on a preliminary literature review. After resolving discrepancies and discussing additional codes, the codebook was revised, and the remainder of the transcripts was coded by a single team member.

The same codebook was applied to the interview transcripts with additional codes added to reflect themes that did not emerge during focus group discussions.

## 3 RESULTS

### 3.1 Focus groups

#### 3.1.1 Participants

Thirty‐four individuals participated in six focus groups at AllianceChicago (three groups), Hines VA (two groups), and the Clinical Directors Network (one group) between September 2017 and March 2018. Key informant interviews were conducted with two clinicians from Rush University Medical Center in lieu of a focus group.

Participants included 14 physicians, 8 nurses, 3 mental health providers, 2 dentists, and 7 medical assistants. Primary clinical areas reported by physicians included pediatrics, family practice, internal medicine, emergency medicine, neurology, ophthalmology, psychiatry, and podiatry.

#### 3.1.2 Role of clinicians in PCT planning

There was emphatic agreement in every group on the importance of engaging clinicians early in research planning. Key themes related to engagement in study planning are summarized in Table 1.

**TABLE 1:** Reasons for engaging clinicians in pragmatic clinical trial planning

Theme	Description	Sample quotes
Identifying the study question	Important to ask clinicians what problems they are trying to solve and identify common issues if studies are to have clinical relevance	*What happens so often when you're taking care of patients, these questions come up all the time‐ Is this the right device? Is this the right dose? …*
Designing the study	Clinicians can provide insight researchers might not be aware of	*I think sometimes studies get designed sort of answering the question, but not really taking into consideration what are the factors… when you're taking care of patients…?*
Understanding the population	Researchers need to be aware of issues related to the specific population such as relevance of the research topic, trust in research, and literacy	*Yeah, people might come in with ideas that are so not even relevant to the population and you're thinking, my God there's all these other things we really need to address, like why this? And then it feels more like an imposition*. *There's a bunch of studies that come our way and a lot of them we end up not participating … it makes our patients feel bad when they can't answer the questions that we give them*. *Looking at it from our experience with patients …I think you would need someone to assist because there's a variety…in terms of the spectrum of literacy*.
Customizing the protocol	Every clinic works differently; researchers need to learn about the constraints and not underestimate the degree of disruption that might be caused	*I mean there are just things that other people don't think about like, oh they're just going to come in and talk to them for two minutes on pain. And then you're like, well, I only have two rooms. That's going to kill my day*. *Just come and talk to us in the before, so that we can talk about these issues instead of just trying to do the implementation and assuming it's all going to work*.
Clinician buy‐in	You cannot stop at getting buy‐in from administrators; clinicians need to believe in the importance of the study so they can translate that to patients and staff	*Being able to have those conversations in really nuanced and sensitive ways and really believing it themselves in order to translate that back to patients would be‐‐that to me would be kind of the trigger on whether or not they will be effective or not*.

#### 3.1.3 PCT case study

When presented with the protocol shown in Figure 1, focus group participants had a generally positive reaction to the limited burden placed on clinicians. However, they also expressed a number of concerns.

Some clinicians working in federally qualified health centers (FQHCs) suggested that patient information in the EHR may not be up to date and thus requires confirmation by a clinician. In contrast, VA providers were confident in EHR data quality and saw no problem with this mode of patient identification. Several participants noted that having clinicians review lists of eligible patients can also be advantageous for researchers:

“Some patients just don't want to be bothered by anything. We would just say don't even bother. You're going to get yelled at.”

Responses were varied regarding permission to contact patients. Most did not think individual permission was needed as long as clinicians are made aware that their patients are participating:

“*I think as long as you're still involving the providers … and you're giving MAs information, you're giving nurses information, because all those people will be getting the questions, and as long as everyone's still educated …and also if I had an opportunity to review the inclusion criteria”*

VA clinicians were generally more comfortable forgoing individual permission and trusted that appropriate review and approval would occur at the institutional level. On the other hand, multiple FQHC providers expressed reluctance to do anything that might interfere with patient‐provider trust:

“Because it often takes a long time for a clinician to gain the trust of patients, right. So if there's going to be something else coming in that might potentially come in the way of that ‐you're not on board with it.”

Regardless of whether permission to contact patients is required, a majority of focus group participants felt that patients would want to make sure their clinician is okay with the study before agreeing to participate. As described by one clinician:

“…I have to reassure [the patient] that…if it was my kid I would do it too, I promise, it's fine. These are good people. I trust them. And that goes a long way because they have relationships with us.”

Finally, there was considerable concern, particularly among FQHC providers, that relying on patients to use technology without assistance for informed consent and data collection would be problematic:

“I mean the whole… issue of phones, or computers, and connectivity, and level of comfort with technology, and level of literacy… it might work with some populations, but I can't see how it would work with us.”

### 3.2 Interviews

#### 3.2.1 Participants

We conducted 18 interviews across 6 CDRNs (Mid‐South, REACHnet, PaTH, LHSnet, OneFlorida, and CAPriCORN) between July and October of 2018. Interview participants included 5 principal investigators, 10 project managers, and 3 participating clinicians.

#### 3.2.2 Patient identification and outreach

ADAPTABLE used the PCORnet Common Data Model to screen for potential study participants within participating CDRNs. Once identified, eligible patients were approached through a combination of “low‐touch” electronic outreach and contact during clinic visits.

Study team members indicated that a small proportion of eligible patients (around 2%‐3%) enrolled in response to electronic outreach without additional follow‐up. During follow‐up phone calls, patients often indicated that they would like to speak to their physician before agreeing to participate in the study. Some interviewees expressed surprise given the “low risk” nature of the intervention. As described by one project manager:

“*I thought when I first started on this aspirin is available over‐the‐counter. People aren't going to be worried about that. But they are. If they've been taking 81 [mg] for 10 years, they're very reluctant to switch unless their doctor tells them it's going to be okay.”*

#### 3.2.3 Clinician buy‐in

At a minimum, it was critical that clinicians were aware that the study was happening and generally supportive. Study team members emphasized the importance of helping clinicians understand the science behind the study question and why it is important for patients. They described multiple approaches to generate support for the study including grand rounds presentations, attendance at faculty and practice team meetings, and one‐on‐one discussions. As described by one interviewee, support for the study by clinicians in leadership positions was also important:

“Having a department chief on board who's going to lend a certain degree of credibility and crack the whip to some extent and say this is something we want to support and then be able to respond to any concerns.”

The importance of site‐level champions was also noted:

“We had permission from the chair and then we had one primary physician who when he was in clinic, his colleagues were more engaged.”

Study team members described the importance of engaging everyone in the clinic, including nursing supervisors, medical assistants, desk attendants, and others. However, it was also noted that the message needs to come from the physician that research is important, and staff are expected to support it.

Team members indicated that, for the most part, they did not encounter resistance to the study from clinicians. Some clinicians were vehement about the choice of aspirin dose but many others did not have a strong opinion. Some clinicians expressed safety concerns for certain patients, but they were typically reassured by explanations of the eligibility criteria and safety measures.

Despite a lack of resistance to the study, clinician motivation to get involved was often low. As described by one team member:

“There wasn't a lot of resistance, but on the flip side there wasn't a lot of buy‐in. They were just like okay, you do it. You can contact my patient.”

Even clinicians with good intentions sometimes had difficulty remaining fully engaged:

It's just that they weren't engaged to the level that they remember to say “you should join this study.” They'd come out and they had forgotten to mention it. They might've gone in intending to mention it.”

#### 3.2.4 Recruitment and enrollment in the clinic setting

**FIGURE 2:**
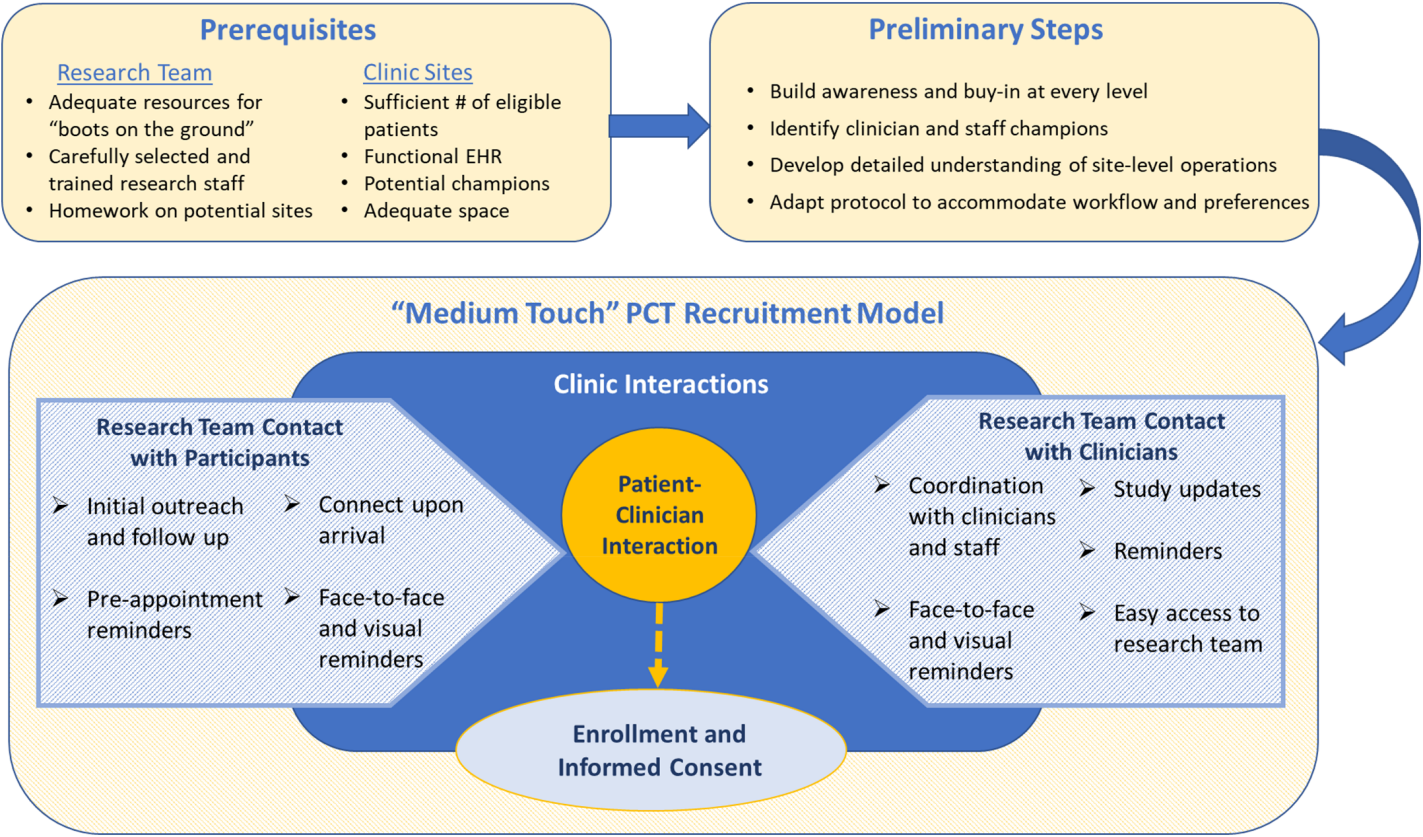
A “Medium‐Touch” approach to engaging clinicians in pragmatic clinical trial Implementation

All of the teams we interviewed described recruitment efforts that took place during clinic visits to supplement low enrollment rates through direct patient outreach. Some indicated that they tried multiple approaches to on‐site recruitment before figuring out what was most effective. Although there is no single model of success, successful strategies involved several key factors related to research team interactions with eligible patients, clinicians, and clinic staff. Keys to success are shown in Figure 2 and described in more detail below.

##### Research team interactions with patients

Most study teams attempted to contact eligible patients in advance of scheduled clinic visits through letters, emails, and/or by phone. This served to “prime” patients, so they were not hearing about the study for the first time in the clinic setting. Providing information about the study and answering questions in advance might also reduce the time required to explain the study during brief clinic visits. One team described using previsit phone calls to ascertain level of interest in the study so they could prioritize in‐person contact with individuals who were interested in participating.

Research coordinators working in clinics were tasked with intercepting eligible patients upon their arrival to remind them about the study and encourage them to discuss the study with their clinician. In some cases patients were handed a study document as a visual reminder to discuss participation and so they would have the information needed to enroll online if enrollment was not completed during the visit.

##### Research team interactions with clinicians

Effectively navigating interactions with clinicians and clinic staff was a common and critical challenge. Interview participants stressed the importance of fostering relationships, establishing friendly rapport with clinic staff, and adapting to the preferences of individual providers. As described by one participant:

“We kind of learned what that provider likes. Sometimes certain providers want us to wait at their workstation so when they come back we're standing right there and they say, ‘okay, go in the room and talk to them’.”

Some noted an initial reluctance to actively engage with physicians:

“I think trying to figure out how we can sell what we're doing…what is the line of how aggressive we should be? Maybe we weren't noisy enough. Just trying to balance our politeness with our assertiveness to go in and be more present.”

The need for high‐quality research coordinators to effectively manage these relationships was a common theme. As described by one project manager:

“We've been very specific on who we bring into clinic because it takes a certain type of personality to be able to do something like this, to be able to multi‐task, to have a good attitude, to work with providers who can sometimes be quite stressed out because of what they have going on.”

Study teams recognized the importance of having clinician support for the study as well as the necessity of keeping the burden placed on clinicians to a minimum:

“I think having the [research] staff take on as much as they possibly can in the process to offload from the clinician because they have so many competing priorities … And that [research] staff are managing everything that they can possibly manage, but also having the support of the clinicians.”

## 4 DISCUSSION

In phase one of this study, focus group discussions with physicians, nurses, and other care providers highlighted the importance of involving front‐line clinicians in PCT planning. PCTs are intended to inform clinical decision‐making, and therefore should address questions that are relevant to clinical practice. Given their motivation to improve patient care, having a say in what research topics are selected is likely to increase clinician willingness to play a role in PCTs. Focus group participants also noted that there are clinicians who are interested in research but do not know how to get involved. Expanded opportunities for clinicians and researchers to collaborate via multiple platforms and modalities were encouraged.

Focus group participants also emphasized the importance of involving clinicians in designing studies that are feasible to implement in the context of clinical care and adapting study protocols to fit workflows that are often unique to a specific clinic. More active involvement by a range of clinical stakeholders in the PCT planning stage may help researchers avoid common barriers to trial implementation. Funding mechanisms that allow adequate time and flexibility for protocol development (eg, the UG3/UH3 model employed for the NIH Health Care Systems Research Collaboratory Demonstration Projects) can allow for more meaningful use of clinician feedback.

Engagement of front‐line clinicians in PCT implementation occurs along a spectrum. At one extreme, “low‐touch” PCTs may involve direct recruitment and enrollment of eligible patients by members of the research team with no need for clinician involvement in delivering the study intervention or conducting follow‐up. A “low‐touch” approach may be viable for PCTs involving a simple, low‐risk intervention where there is a large population of eligible patients, an electronic infrastructure to facilitate patient outreach, and a willingness to accept a very low rate of enrollment. Based on their experience recruiting for the ADAPTABLE trial, Pfaff et al^18^ suggest a combined approach that includes electronic and in‐clinic recruitment and recommend future work to understand the demographic skew that results from electronic recruitment approaches.

At the other end of the spectrum, “high‐touch” PCTs can be defined as those in which clinicians play an active role in any or all of the following: identification of eligible patients, recruitment and enrollment, delivery of the study intervention, and data collection and participant follow‐up. A “high‐touch” approach may be necessary when there are a limited number of eligible patients, barriers to identifying patients via EHR, institutional policies that limit researchers' direct access to eligible patients, a patient population that requires extra support to participate, or complex study interventions.

We propose that many PCTs, such as ADAPTABLE, can be categorized as “medium‐touch” with regard to clinician involvement. A “medium‐touch” approach works best when there are an adequate number of eligible patients at participating sites, an EHR that is functional for identifying eligible patients, site‐level study champions, and adequate space to accommodate research activities without impeding clinic workflow. The optimal research team for this type of trial has adequate resources to provide “boots on the ground” and carefully selected and trained research staff (eg, those with adequate emotional maturity and relationship‐building skills). Successful trial implementation is more likely when researchers take the time to build awareness and buy‐in at every level of the organization, identify clinician and staff champions, develop a detailed understanding of site‐level operations, and adapt study protocols to accommodate individual preferences and workflow.

With increasingly high demands on clinician time and the fact that their first priority is, as it should be, providing the best possible patient care, research teams should take on as much of the burden of trial implementation as possible. A “medium‐touch” approach to recruitment and enrollment is grounded in the idea that clinician effort should be focused where it is most critical—to reassure eligible patients that trial participation is a safe alternative for them. A common theme from both focus group and interview participants was that patients place a lot of trust in their clinicians. Thus, as is the case for traditional randomized clinical trials, clinician support may be a critical element of successful PCT recruitment and protocols that optimize this role while limiting clinician burden may be the most successful.

With the increasing focus on learning health systems as a nexus for real‐world research, the need for optimization of PCTs will only grow in importance. We have attempted to offer solutions to some common barriers to engaging clinicians in PCTs but given that this is a relatively nascent addition to the research landscape, many questions remain. Ongoing and recently completed trials may provide additional insights regarding successful and unsuccessful strategies for PCT implementation and efforts to capture these learnings such as the NIH Collaboratory Living Textbook of Pragmatic Clinical Trials remain critical.^19^ As noted in a recent article by Simon et al,^20^ the goal of a health care system where the vast majority of clinical decisions are based on reliable evidence can only be achieved with commitment by researchers, health system leaders, research funders, and regulators in partnership with patients, caregivers, and clinicians.

## CONFLICT OF INTEREST

The authors have no conflicts of interest to declare.
